# The cat is out of the bag – point-of-care testing (POCT) is here to stay

**DOI:** 10.2807/1560-7917.ES.2020.25.44.2001854

**Published:** 2020-11-05

**Authors:** Nick Phin, Susan M Poutanen

**Affiliations:** 1 Public Health England (PHE), Colindale, London, United Kingdom; 2 University of Chester, Chester, United Kingdom; 3 University Health Network/Sinai Health Department of Microbiology, Toronto, Canada; 4 Department of Laboratory Medicine and Pathobiology, Department of Medicine, University of Toronto, Toronto, Canada

**Keywords:** point-of-care testing, sensitivity, specificity, laboratory surveillance, public health, patient management

Recently, a friend had to take their cat to the veterinary surgeon as she was out of sorts. After a thorough examination, the veterinarian felt that a haematology and biochemistry screen would be helpful in excluding some of the less obvious conditions that could be present. The friend agreed and asked if they should call back for the results at the end of the week, to which he responded that if they can hold on for 10 minutes, the two of them could discuss the results and any future management coming out of them. We were astonished and wondered why currently such service seemed to be easier to access for animals than for humans.

Point-of-care tests (POCTs) have increased in number in recent years in parallel to other technological advances. And, more than ever before, the current coronavirus disease (COVID-19) pandemic has brought the need for the widespread adoption of sensitive and specific POCTs to the fore. The diagnosis of COVID-19 in the absence of specific symptoms, a robust and consistent clinical syndrome and high rates of asymptomatic infections relies on testing to identify cases and enable the institution of appropriate clinical and public health measures. A POCT performed at, or near the point of patient care, can improve accessibility, provide timely advice, allow for immediate action and increase the likelihood of those infected adopting self-isolation from the start.

The eight articles in this special issue of *Eurosurveillance* highlight the benefits of POCT and identify issues that need to be taken into account when they are introduced into a clinical setting or implemented as part of surveillance that may form the basis for public health action.

The paper by Fernàndez-López et al. [1] describes an assessment of a community-based HIV testing service in Catalonia, Spain, that used POCT to increase the availability and accessibility of testing, particularly opportunistic testing. During the period of the assessment, from 1995 to 2018, the researchers were able to show a substantial increase in the number of tests undertaken particularly in people who inject drugs, a group that can be hard to engage with, and many cases were subsequently linked to care. In addition, the increased accessibility and uptake of testing provided some reassurance that the fall in cases seen following the peak in 2014, was likely real, and not a consequence of under-ascertainment.

Celma et al. [2] illustrate the importance of recognising the limitations of POCT and the need to understand the potential for false-positive and false-negative test results, which will vary depending on the characteristics of the POCT and the prevalence of the disease in the population. In their study, POCTs were placed within England’s National Health Service hospital laboratories as opposed to near patient care. The positive predictive value of the most commonly used rotavirus immunochromoatography-based rapid test in their study varied from only 21.4% from July to November when rotavirus is less prevalent, to 89.6% during January to April, when rotavirus is more prevalent in the temperate northern hemisphere. This illustrates the importance of implementing confirmatory testing given the implications that false-positive rotavirus test results may have on both patient management and national surveillance.

Two articles focus on the use of POCT to tackle the rise in antimicrobial resistance (AMR) in *Neisseria gonorrhoeae*, an increasing public health concern. Vegvari et al. [3] provide a model of using POCT to inform treatment choice for *N. gonorrhoeae* by detecting stepping-stone resistance determinants relevant to the selection of resistance to gepotidacin, a novel antibiotic (type II topoisomerase inhibitor) still used only in clinical trials. The potential for POCT to be used to reduce the potential for selection of resistance is a welcome application in the era of increasing AMR. Knowledge of baseline resistance-determinant rates and rates of mutations with and without pressure from the antimicrobial of interest are needed to determine the usefulness of this approach and the POCT test characteristics would need to be taken into account.

In a further paper, Harding-Esch et al. [4] analyse the benefits and costs of five antimicrobial resistant *N. gonorrhoeae* POCT strategies and their impact on treatment optimisation, reducing selection pressures on a key antibiotic and cost. They apply a simulation model using a cohort of patients infected with *N. gonorrhoea*e, the majority of whom were men who have sex with men. Primary outcomes were: total costs; percentage of people given optimal treatment; percentage of people given non-ceftriaxone optimal treatment; cost-effectiveness. The authors found that all AMR-POCT strategies can enable correct antibiotic therapy at diagnosis and improve antibiotic stewardship but cost more than standard treatment and would require investment. This has the potential to be mitigated if a small reduction in test costs could be achieved. In addition, the different strategies had differing trade-offs with respect to avoiding suboptimal treatments, costs and ceftriaxone-sparing treatment. This highlights the importance of clearly setting out and agreeing on AMR-POCT programme objectives.

The uptake of recommendations to incorporate C-reactive protein (CRP) POCT in clinical practice was assessed by means of a McNulty–Zelen cluster pragmatic randomised clinical trial by Eley et al. [5]. They noted a non-significant reduction in the odds of prescribing antimicrobials for cough in those with access to CRP POCT, but also noted variable uptake of the POCT in participating practices despite National Institute for Health and Care Excellence (NICE) guidance supporting its use. Furthermore, there was variable uptake of NICE recommendations for delayed treatment for patients with CRP levels between 20 and 100 mg/L. Unlike the potential to implement change within laboratory settings where there is a finite number of laboratories that are accredited and subject to licensing, full impact of POCT, if implemented as part of a national guidance, may not be put in practice given the challenge of influencing adoption by a much large number of independent clinics and clinicians.

Three papers in the special issue are dealing with POCT to detect respiratory viruses, in particular influenza. Schneider et al. [6] report on their experience with POCT for influenza A, influenza B, and respiratory syncytial virus in all hospital emergency departments in the capital region of Denmark. They noted a reduction in antimicrobial use and reduction in hospitalisation days, and an increase in antiviral use in patients with positive POCT results but no difference in mortality or readmission rates. Notwithstanding the limitation of not having a control group of people tested with traditional non-POCT, the authors note the potential for benefit from respiratory syndromic POCT. They ensured all POCT results were reported into their national microbiology database, but acknowledged the potential for impact on their national surveillance system, given the lack of subtyping available.

In a second paper, Dickson et al. [7] provide an assessment of the impact and effectiveness of national data capture following the use of influenza POCT introduced to aid patient triage during two consecutive influenza seasons. The authors from Scotland found that the areas using POCT increased over the two seasons, the capture of positive POCT results improved between seasons but recording of negative results was incomplete. While there was a clear benefit to patient management, the authors note that the greatest challenge is capturing data for national surveillance. They specifically mention the lack of universal instrument interfaces to laboratory information management systems with the resultant potential for lack of reporting and transcription errors. They also note challenges with documentation of results as belonging to POCT with the potential for misclassification of testing type.

Finally, a perspective paper by Dickson et al. [8] highlights the opportunities and challenges that influenza POCT presents, and describes potential solutions. Challenges highlighted by the authors include integration into clinical workflow, standardisation of protocols, data procurement for surveillance purposes, and characterisation of viral isolates. To address the latter two, the authors specifically recommend consideration of technological solutions to facilitate upload of POCT data to cloud databases to enable data capture in surveillance systems and recommend a national policy for procurement of specimens to enable influenza subtyping, sequencing, and antiviral susceptibility testing.

Molecular and genetic insights and technological advances in computing and miniaturisation have meant that tests with high sensitivity and specificity hitherto considered the domain of reference laboratories are now easily accessible to front-line clinicians and non-traditional settings such as pharmacies. The availability of such tests, at the point of care, are transforming the way medicine is practised. Being able to identify, as part of a consultation, that a patient has a particular infection or condition, allows the clinician to provide the patient tailored treatment and advice in real-time. This has also the potential to achieve a number of other benefits – antibiotic stewardship by only using antibiotics where a bacterial infection is present and only those specific to the infection, patient education with advice specific to the infection, addressing public health issues by advising on contact tracing, cost savings and better utilisation of clinician and patient time by getting treatment right from the start.

However, limitations of POCT need to be recognised. Not all POCTs are equal in performance with some having lower sensitivity and specificity than others. Recognising the potential impact of these test characteristics on false-positive and false-negative results and the need for confirmatory testing is important. Training POCT users in the correct use and quality oversight of POCT is challenging given the diverse number of clinicians and locations involved with POCT. Integrating POCT data and submitting specimens for surveillance purposes poses additional challenges with public health implications. Policies and regulatory oversight of POCT implementation are important to assure that these limitations are addressed and to ensure that the full benefits of POCT are realised without incurring harm. The cat is already out of the bag – it behoves us to tame it.
